# Direct programming of human pluripotent stem cells into endothelial progenitors with SOX17 and FGF2

**DOI:** 10.1016/j.stemcr.2024.02.006

**Published:** 2024-03-21

**Authors:** Michael W. Ream, Lauren N. Randolph, Yuqian Jiang, Yun Chang, Xiaoping Bao, Xiaojun Lance Lian

**Affiliations:** 1 Department of Biomedical Engineering, Pennsylvania State University, University Park, PA 16802, USA; 2 Department of Biology, Pennsylvania State University, University Park, PA 16802, USA; 3 The Huck Institutes of the Life Sciences, Pennsylvania State University, University Park, PA 16802, USA; 4 Davidson School of Chemical Engineering, Purdue University, West Lafayette, IN 47907, USA

## Abstract

Transcription factors (TFs) are pivotal in guiding stem cell behavior, including their maintenance and differentiation. Using single-cell RNA sequencing, we investigated TFs expressed in endothelial progenitors (EPs) derived from human pluripotent stem cells (hPSCs) and identified upregulated expression of SOXF factors *SOX7*, *SOX17*, and *SOX18* in the EP population. To test whether overexpression of these factors increases differentiation efficiency, we established inducible hPSC lines for each SOXF factor and found only SOX17 overexpression robustly increased the percentage of cells expressing CD34 and vascular endothelial cadherin (VEC). Conversely, *SOX17* knockdown via CRISPR-Cas13d significantly compromised EP differentiation. Intriguingly, we discovered *SOX17* overexpression alone was sufficient to generate CD34^+^VEC^+^CD31^−^ cells, and, when combined with FGF2 treatment, more than 90% of CD34^+^VEC^+^CD31^+^ EP was produced. These cells are capable of further differentiating into endothelial cells. These findings underscore an undiscovered role of SOX17 in programming hPSCs toward an EP lineage, illuminating pivotal mechanisms in EP differentiation.

## Highlights

• scRNA-seq analysis of EPs identifies differential expression of three SOXF factors
• Overexpression of SOX17 can enhance CHIR99021-mediated EP differentiation
• Overexpression of SOX17 alone is sufficient to generate 50% CD34^+^VEC^+^ EPs
• FGF2 can enhance EP programming via SOX17, yielding 90% CD31^+^CD34^+^VEC^+^ EPs

## Graphical abstract

**Figure:**
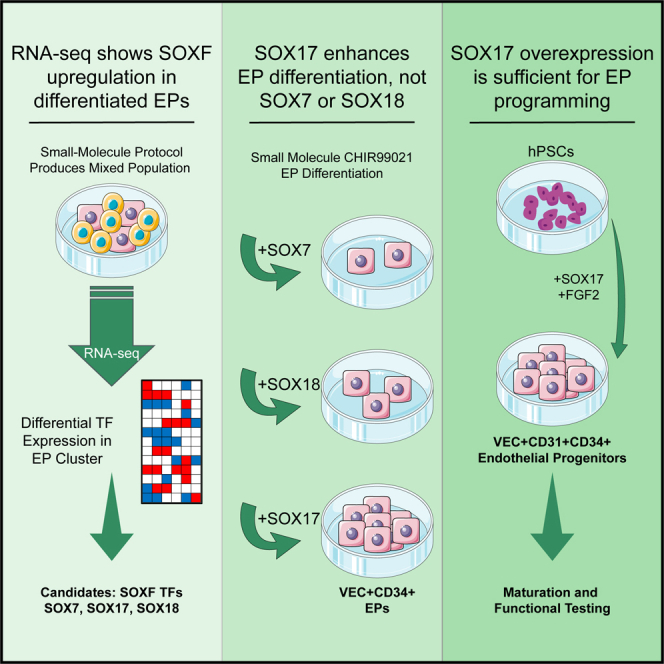

## Teaser

In this article, Lian and colleagues reveal the pivotal role of SOX17 in programming hPSCs toward an EP lineage. Through scRNA-seq, they identify elevated expression of SOXF factors in EPs derived from hPSCs. Overexpression of SOX17 alone enables EP programming from hPSCs. Combined with FGF2 treatment, this approach yields more than 90% CD34^+^VEC^+^CD31^+^ EPs, unveiling a novel SOX17 programming role.

## Introduction

Generating homogeneous populations of clinically relevant cell types from human pluripotent stem cells (hPSCs) can be challenging when using growth factors and small molecules, as this approach often leads to the formation of heterogeneous cell populations (Choi et al., 2012; Ditadi et al., 2015; Lian et al., 2014; Pagliuca et al., 2014). In the past, the differentiation of hPSCs was often achieved through the formation of embryoid bodies (EBs). A study by Levenberg et al. showed that the absence of self-renewal factors during EB formation led to spontaneous differentiation of EPs from hPSCs, albeit with very low efficiency (<2%) (Levenberg et al., 2002). To improve the efficiency of EP differentiation from hPSCs, researchers have been exploring alternative methods. One such method involves using growth factors that enhance EP development, as well as 2D surface culturing of hPSCs without EB formation. Typically, hPSCs are first differentiated into mesodermal cells, which can then be further differentiated into EPs. In 2014, Lian et al. showed that a GSK3β inhibitor can be used to generate mesodermal cells quickly and efficiently from hPSCs, with over 95% efficiency in just 2 days. The resulting mesodermal cells can be cultured in a basal medium with VEGF treatment for an additional 3–4 days to generate EPs, which express CD31, CD34, and vascular endothelial cadherin (VEC), with an efficiency of approximately 20% (Lian et al., 2014). To further improve the efficiency of this process, researchers have incorporated bone morphogenetic protein 4 (BMP4) and activin A along with CHIR99021 during mesoderm differentiation (Zhang et al., 2017), and included an adenylyl cyclase activator during the mesoderm-to-EP differentiation stage. These modifications have increased the efficiency of EP differentiation to 61% (Patsch et al., 2015).

In addition to directed differentiation via small molecules and growth factors, another approach to generate desired cell types from hPSCs is forward programming. This method involves overexpression of specific transcription factors (TFs) in hPSCs to promote direct conversion into the desired cell types, bypassing relevant developmental stages. Forward programming has the potential to generate populations of desired cells with high efficiency in a shorter time frame. For example, overexpression of *NFIA* is sufficient to generate astrocytes from neural stem cells within 3 weeks, compared with 3–6 months required by growth factor and small molecule-based protocols (Tchieu et al., 2019).

To apply forward programming to derive EPs from hPSCs, it is essential to identify the TFs specifically expressed in this population. Recent advances in single-cell analysis techniques have enabled researchers to characterize differentiated cell types with unprecedented depth. Single-cell RNA sequencing (scRNA-seq) analysis has revealed heterogeneity in many differentiated populations, including endothelial cells (ECs) and pancreatic beta cells (Paik et al., 2018; Veres et al., 2019). This approach can be used to identify critical TFs solely expressed in desired cell populations, which may be candidates for forward programming. Using scRNA-seq analysis, we identified SOXF factors (Lefebvre et al., 2007), SOX7, SOX17, and SOX18*,* as specifically expressed in our hPSC-derived EPs. We systematically studied SOX17 during hPSC differentiation to EPs and found that SOX17 overexpression enhances differentiation efficiency. Knockdown of *SOX17* via CRISPR-Cas13d, however, inhibited EP differentiation. Importantly, overexpression of SOX17 alone is sufficient to program hPSCs into CD34^+^VEC^+^CD31^−^ cells. After screening several growth factors, we discovered that fibroblast growth factor 2 (FGF2) treatment enabled SOX17-programmed cells to become CD34^+^VEC^+^CD31^+^ EPs.

Our findings provide new insights into endothelial development and highlight a novel role for SOX17 in deriving EPs from hPSCs. This discovery enables a unique EP programming method via overexpression of SOX17.

## Results

### scRNA-seq reveals SOXF factors expression in EPs

**Figure 1:**
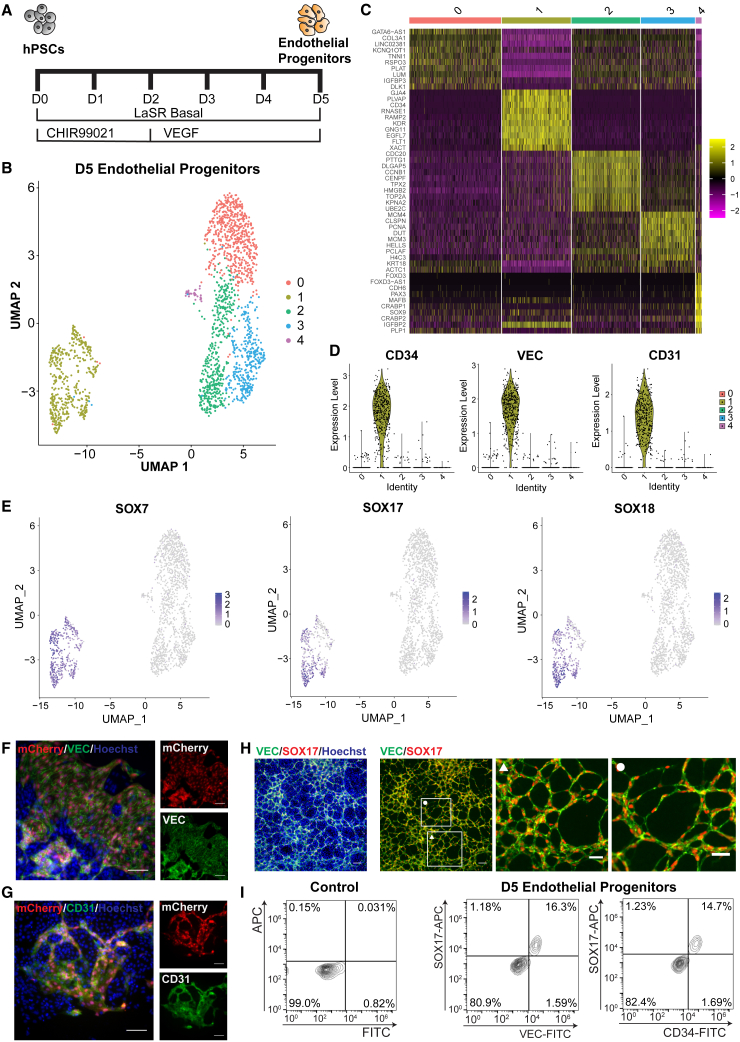
CH-induced differentiation method yields EP population expressing endothelial markers and SOXF family (A) Schematic of EP differentiation. (B) UMAP dimensional reduction projection showing clustering of cells produced after 5 days of differentiation. (C) Heatmap indicating the top 10 differentially expressed genes for each cluster. (D) Violin plots identifying CD34, VEC, and CD31 expression in cluster 1. (E) SOXF family member expression in cluster 1 population. (F and G) Immunofluorescence images of day 5 EPs derived from SOX17-mCherry reporter H9 cells. mCherry expression is seen in cells that express VEC (F) and CD31 (G). Nuclear staining was done with Hoechst 33342. Scale bars, 100 μm. (H) Immunofluorescent images showing VEC and SOX17 co-expression in day 5 cells differentiated from 6-9-9 cells. White boxes indicate locations of enlarged views with symbols indicating the corresponding image. Nuclear staining was done with Hoechst 33342. Left two scale bars are 100 μm and right two scale bars are 50 μm. (I) Flow cytometry analysis showing co-expression of SOX17 and VEC or SOX17 and CD34 in day 5 cells differentiated from 6-9-9 cells.

We developed a small molecule-based protocol (Bao et al., 2015; Lian et al., 2014; Randolph et al., 2019) to generate EPs from hPSCs using an initial pulse of Wnt/β-catenin signaling activation with a GSK3β inhibitor, CHIR99021 (CH). These EPs express CD34, CD31, and VEC and generate primitive vascular structures (Galat et al., 2017; Lian et al., 2014). To identify TFs specifically enriched in the EP population, we performed scRNA-seq analysis of day 5 differentiated cells (Figure 1A). We sequenced 2,673 cells and after quality control filters were applied, we included 1,917 cells in our analysis (Figure S1A). Dimensional reduction and supervised clustering showed five distinct clusters of cells on day 5 of differentiation (Figures 1B, 1C, S1B, and S1D). Clusters (0, 1, 2, 3, and 4) were composed of 609, 457, 454, 358, and 39 cells, respectively. Based on the top 100 differentially expressed genes, cluster-0 likely represents cardiac progenitors with upregulation of *TNNI1*, *HAND1*, and *TMEM88* (Palpant et al., 2013) (Figure 1C, Table S1). Cluster-3 cells show differential expression of *MYL7*, *MYL9*, and *HAND1*, and, therefore, may be labeled as atrial cardiac progenitors (Table S1). Cluster-4 has increased expression of *SOX2* and *POU5F1* and may represent residual undifferentiated hPSCs (Table S1). In the UMAP projection, cluster-1 was distinctly separated from the other four clusters, and exhibited elevated levels of CD34, CDH5 (VEC), and PECAM1 (CD31), indicating its EP identity. Additionally, other genes that have been identified as critical regulators for endothelial lineage development, including *ETS1*, *MECOM*, *CD93*, and *KDR*, showed increased expression in cluster-1 as compared with all other clusters (Figure S1E).

Inspection of differentially expressed TFs uncovered high expression of all three SOXF factors (Figure 1E) in the EP cell cluster (cluster-1). No other SOX factors were differentially expressed in EPs (cluster-1) (Table S1). To confirm SOXF expression in our EPs, we used a SOX17-mCherry knockin reporter hPSC line (Ng et al., 2016) and differentiated the knockin hPSCs to day 5 differentiated cells. The reporter cell line was validated by antibody staining on day 5 of differentiation (Figure S1F). In addition, mCherry expression only occurred in cells also expressing VEC and CD31 on day 5 of differentiation (Figures 1F and 1G). The co-expression of SOX17 with EP markers VEC and CD34 was also observed in an additional hPSC line (6-9-9 induced pluripotent stem cell [iPSC] line) by immunostaining and flow cytometry (Figures 1H and 1I). These data provide strong evidence that our Wnt activation protocol does result in EPs, and our scRNA-seq data revealed upregulated expression of SOXF factors in this population.

### Overexpression of SOX17 but not SOX7 or SOX18 enhances EP differentiation

**Figure 2:**
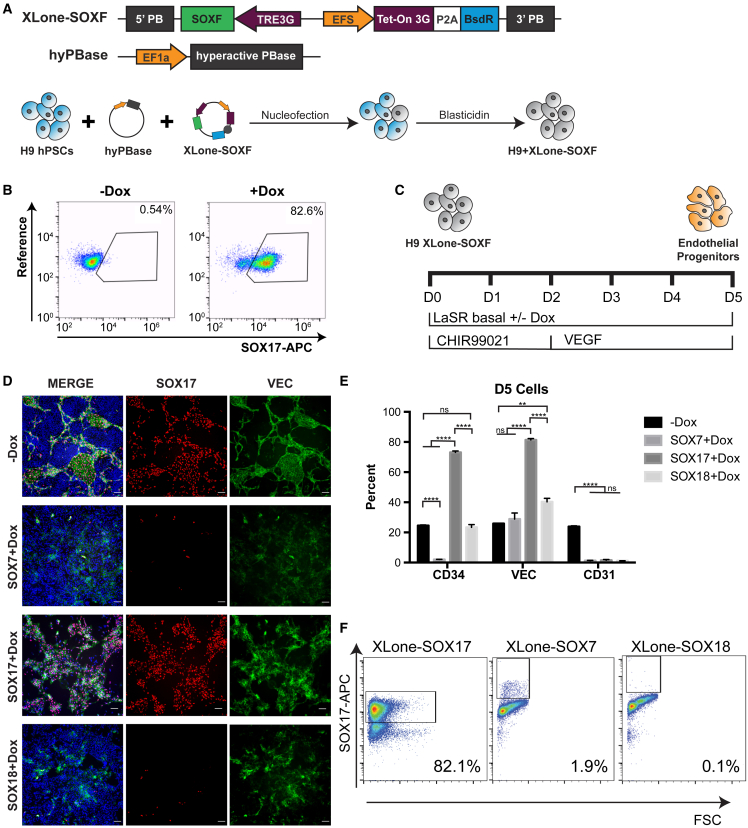
SOX17 is the only SOXF member that increases EP differentiation efficiency when overexpressed (A) Schematic showing the generation of XLone-SOXF (SOX7, SOX17, or SOX18) hPSCs. (B) Flow cytometry analysis of validating inducible SOX17 expression in XLone-SOX17 H9 cells treated with or without Dox for 24 h. (C) Schematic illustrating culture conditions for SOXF overexpression during EP differentiation. (D) Immunofluorescence analysis of day 5 cells differentiated with Dox for each XLone-SOXF cell line and control without Dox. Cells were stained with SOX17 and VEC antibodies and nuclear stain (Hoechst 33342). Scale bars, 100 μm. (E) Quantification of flow cytometry analysis of CD34, VEC, and CD31 expression in day 5 XLone-SOXF H9 cells treated with Dox and control without Dox (n = 3, independent experiments). ^∗∗^p < 0.01; ^∗∗∗∗^p < 0.0001. Error bars represent SEM. (F) Flow cytometry analysis of SOX17 expression in day 5 XLone-SOX17, Xlone-SOX7, and Xlone-SOX18 H9 cells differentiated with Dox.

Upon discovering that all three SOXF factors are expressed in our EPs, we sought to understand which, if any, of these factors play a functional role in determining this cell fate. To address this question, we generated cell lines with inducible overexpression of *SOX7*, *SOX17*, and *SOX18* by cloning each TF into our doxycycline (Dox)-inducible, PiggyBac-based XLone construct (Randolph et al., 2017) (Figure 2A). This construct was then introduced into hPSCs, and cells successfully incorporating the construct were purified by drug selection (Figure 2A). The modified cells were then referred to as XLone-SOX7, Xlone-SOX17, and XLone-SOX18 H9 cells. To confirm the intended transgene expression of the resulting cells, we treated XLone-SOX17 H9 cells with or without Dox for 24 h. This treatment resulted in a strong overexpression of SOX17 in 82.6% of cells. Importantly, we observed negligible expression in cells without Dox treatment (Figure 2B). This was consistent with our previous findings for XLone and indicates the successful generation of stable transgenic XLone-SOXF cell lines.

To test whether overexpression of SOXF factors enhances Wnt activation-induced EP differentiation, we differentiated each cell line to EPs using CH and vascular endothelial growth factor (VEGF) in the presence or absence of Dox and compared the expression of CD34, VEC, and CD31 (Figures 2C and S2A). The expression levels of these markers in cells without Dox treatment was consistent with our earlier experiments, and the differentiation efficiency was comparable with our previous results (Lian et al., 2014; Randolph et al., 2019) (Figure 2D, 2E, and S2B). We observed a significant increase in the SOX17^+^ population, up to 82%, in cells differentiated in the presence of Dox for XLone-SOX17 H9 cells, as expected. In contrast, there were few SOX17^+^ cells in the day 5 cells generated from either XLone-SOX7 or XLone-SOX18 H9 cells (Figures 2D and 2F). XLone-SOX7 H9 cells showed a significant decrease in the percentage of CD34^+^ cells with no change in the percentage of VEC^+^ cells (Figures 2D, 2E, and S2B). XLone-SOX18 H9 cells did not exhibit any change in the CD34^+^ population, but showed a statistically significant increase in the VEC^+^ population (40.27% ± 2.32%, p = 0.0035) (Figures 2D, 2E, and S2B). The day 5 cells resulting from XLone-SOX17 H9 cells showed significant increases in CD34^+^ (73.10% ± 0.94%, p = 1.15e−6) and VEC^+^ (81.40% ± 0.90%, p = 4.97e−7) populations compared with differentiation without any transgene overexpression (Figures 2D, 2E, and S2B). Fluorescent microscopy revealed that the majority of SOX17^+^ cells were also VEC^+^ (Figure 2D). Interestingly, the overexpression of each SOXF factor resulted in a loss of CD31 expression (Figures 2E and S2B). Collectively, this demonstrates that forced overexpression of *SOX17*, but not *SOX7* or *SOX18*, increases Wnt activation-induced generation of CD34^+^VEC^+^ progenitors, highlighting a functional role for *SOX17* in the acquisition of EC fate.

### SOX17 expression occurs prior to EP markers and Cas13d interference with SOX17 inhibits EP differentiation

**Figure 3:**
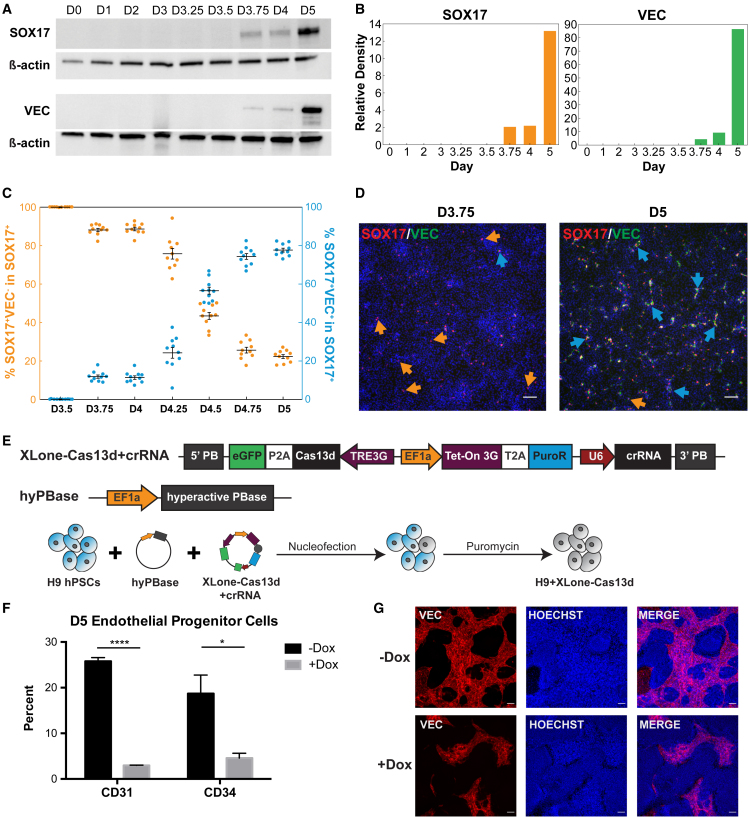
SOX17 expression occurs immediately prior to endothelial marker expression and transcriptional interference reduces endothelial marker expression (A) Western blots showing SOX17 and VEC protein levels over the course of differentiation. Β-actin was used as a housekeeping gene. (B) Quantification of blots shown in (A) normalized to β-actin. (C) Quantification of SOX17^+^VEC^−^ cells (left axis, orange) and SOX17^+^VEC^+^ cells (right axis, blue) in the SOX17^+^ population as derived from analysis of immunofluorescent images (n = 10, independent experiments). Error bars represent SEM. (D) Representative immunofluorescence images used for quantification in (C) for day 3.75 and day 5 (n = 10). Orange arrows highlight SOX17^+^VEC^−^ cells and blue arrows highlight SOX17^+^VEC^+^ cells. Nuclear staining was done with Hoechst 33342. Scale bars, 100 μm. (E) Schematic illustrating the generation of a cell line with Cas13d-based inducible SOX17 knockdown. (F) Quantification of flow cytometry experiments analyzing the change in size of the day 5 population expressing CD31 or CD34 for cells treated with and without Dox (n = 3, independent experiments). ^∗^p < 0.05; ^∗∗∗∗^p < 0.0001. Error bars represent SEM. (G) Immunofluorescence images of D5 cells differentiated with or without Dox stained with VEC. Nuclear staining was done with Hoechst 33342. Scale bars, 100 μm.

To better understand the role of SOX17 during EP differentiation, we characterized SOX17 expression kinetics along hPSC differentiation to EPs. We differentiated hPSCs following the Wnt activation protocol illustrated in Figure 1A and collected cells daily until day 3 and every 6 h after day 3 until day 5. Western blot analysis showed SOX17 and VEC are first detected on day 3.75 (Figures 3A and 3B). Immunofluorescent analysis revealed there are more cells expressing SOX17 alone on day 3.75, and this SOX17 single-positive cell number gradually decreases as cells become double-positive for SOX17 and VEC over time (Figures 3C, 3D, and S2C). These data indicate that expression of SOX17 occurred before the appearance of VEC^+^ cells.

To further investigate the role of SOX17 in our EP differentiation, we performed a loss-of-function analysis using a Cas13d-mediated knockdown approach. Cas13d is a member of the Cas13 family that can efficiently knockdown mRNA transcripts (Konermann et al., 2018). We cloned Cas13d into our XLone plasmid construct, driven by the inducible TRE3G promoter (Randolph et al., 2017) (Figure 3E). Additionally, we incorporated a U6 promoter expressing SOX17 crRNA into the plasmid, creating a single transposon system for Cas13d interference. We transfected this plasmid into H9 cells and used puromycin drug selection to purify the cells that integrated the XLone-Cas13d system (Figure 3E). Flow cytometry analysis confirmed robust and near complete SOX17 knockdown efficiency via Cas13d during definitive endoderm differentiation with or without Dox treatment (Figure S2D). EP differentiation using this cell line with Dox treatment showed abrogation of CD31 and CD34 expression, as well as decreased VEC expression upon SOX17 knockdown (Figures 3F, 3G, and S2E). These results suggest that SOX17 expression is necessary for the formation of EPs from hPSCs.

### SOX17 is sufficient to program hPSCs into CD34^+^VEC^+^ cells

**Figure 4:**
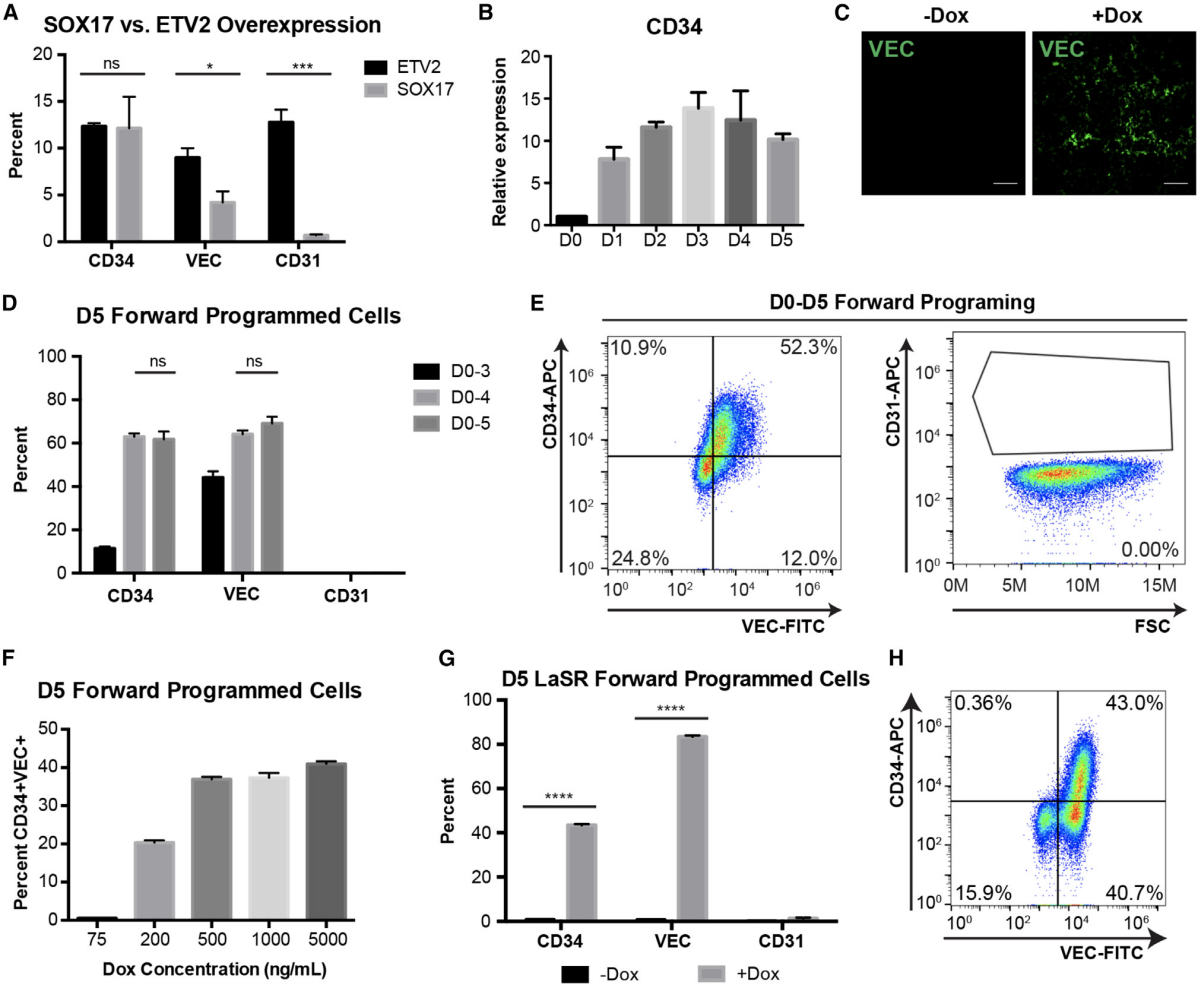
SOX17 forward programming is sufficient to produce CD34^+^VEC^+^ cells (A) Quantification of flow cytometry analysis of day 5 SOX17 or ETV2 forward programmed cells for EP markers VEC, CD34, and CD31. (n = 3, independent experiments) ∗p < 0.05. ^∗∗∗^p < 0.001. ns, not significant. (B) qPCR analysis of CD34 expression from day 0 to 5 for LaSR Basal + Dox condition. n = 3, technical replicates. (C) Immunofluorescence analysis of VEC expression in day 5 cells for each condition. Scale bars, 130 μm. (D) Quantification of flow cytometry analysis for day 5 cells for EP markers CD34, VEC, and CD31 for cells treated with Dox for 3, 4, and 5 days (n = 3, independent experiments). (E) Representative flow cytometry plots showing SOX17 forward programmed cells with Dox treatment for 5 days. (F) Quantification of flow cytometry analysis for different Dox concentrations used for 5 days of treatment (n = 3, independent experiments). (G) Quantification of day 5 flow cytometry data for day 5 cells cultured in LaSR hPSC media with or without Dox. (n = 3, independent experiments.) ^∗∗∗∗^p < 0.0001. (H) Representative flow cytometry analysis of day 5 cells treated with or without Dox in LaSR hPSC media.

To determine if SOX17 overexpression can directly convert hPSCs into EPs without CH and VEGF, we subjected XLone-SOX17 H9 cells to a basal medium (LaSR basal), with or without Dox treatment. As expected, day 5 cells cultured in the basal medium without Dox did not turn on expression of CD34, CD31, or VEC. However, when Dox treatment is applied, a population of the cells show expression of CD34 and VEC, with CD34 mRNA expression increasing as early as day 1 (Figures 4A–4C). We also used the XLone-ETV2 line as a positive control for hPSC programming, given that ETV2 is known to enable EP programming of hPSCs (Wang et al., 2020). Notably, SOX17 produces a similar percentage of CD34-expressing cells as ETV2 (Figure 4A).

To confirm that SOX17-mediated forward programming was not cell line dependent, we generated and validated XLone-SOX17 cell lines using another hPSC line (H1 OCT4-GFP cells) (Figures S3A and S3B). Cells were then cultured with or without Dox treatment for 3 days in LaSR basal media (Figure S3C). We confirmed the loss of pluripotency by decrease in the GFP expression for XLone-SOX17 H1 OCT4-GFP cells from 97.1% on day 0 to 20.8% on day 5 (Figures S3D and S3F). Remarkably, in the presence of Dox, more than 85% of cells expressed VEC by day 5. In contrast, without Dox, none of the cells expressed VEC (Figures S3E and S3F). However, the percentage of CD34^+^ cells is less than 10% with Dox treatment, which is relatively low (Figure S3F).

To further enhance the efficiency of SOX17 forward programming, we speculated that cell density during differentiation and the duration of Dox treatment might influence efficiency. We programmed hPSCs and, on day 2, adjusted their density by passaging. We also varied the Dox treatment duration between 3 and 5 days. With the day 2 passaging, a longer Dox duration (4–5 days) significantly boosted both the CD34^+^ and VEC^+^ populations compared with a 3-day treatment (Figures 4D and S4A). Regarding the impact of adjusting cell density, the VEC^+^ population increased from less than 5% (without day 2 passaging) to 44.2% ± 2.8% (with day 2 passaging) using 3-day Dox treatment (Figures 4A–4D, S4A). A longer than 3-day duration of Dox treatment increased the efficiency to 51% ± 2.3% CD34^+^VEC^+^ with no significant difference between 4 or 5 days of Dox treatment (CD34 p = 0.79, VEC p = 0.23) (Figures 4D, 4E, and S4A). This demonstrates that extended duration of SOX17 overexpression for more than 3 days and efforts to maintain lower cell density increase the efficiency of *SOX17* forward programming. We reasoned that passaging on day 2 might either select for cells undergoing forward programming or that altering cell-to-cell contact could boost differentiation. After optimizing the required duration of Dox treatment, we tested a range of concentrations to determine the optimal level of transgene activation (Randolph et al., 2017). We found that lower Dox concentrations (75 and 200 ng/mL) resulted in a decreased CD34^+^VEC^+^ population and that at least 500 ng/mL Dox was required to achieve maximal efficiency (Figures 4F and S4B). Based on our prior characterizations (Randolph et al., 2017), this indicates that maximal transgene expression is required for *SOX17* forward programming. We replicated this finding with another hPSC line (6-9-9 XLone-SOX17) and obtained 57.2% ± 2.8% of the resulting cells expressing both CD34 and VEC (Figure S4C).

In an effort to examine the potency of SOX17 as a mediator of forward programming, we investigated whether hPSCs could be programmed through SOX17 when cultured in pluripotency maintenance media, rather than in basal media. Cells were cultured in one of two pluripotency maintenance hPSC media, LaSR or TeSR, in the presence or absence of Dox (Figures S5A and S5B). *SOX17* forward programming was able to overcome pluripotency maintenance signals in hPSC media and cause cells to differentiate as evidenced by loss of pluripotent morphology (Figures S5A and S5B). Day 5 flow cytometry analysis showed the presence of CD34^+^VEC^+^ cells and CD34^−^VEC^+^ cells (Figures 4G, 4H, S5C, and S5D). We did not observe expression of CD31 in the day 5 cells (Figures 4G, 4H, and S5D). Both hPSC media produced CD34^+^VEC^+^ cells and CD34^−^VEC^+^ cells, albeit at different efficiencies (Figures 4H and S5C). This could be due to the increased amount of bovine serum albumin in TeSR as compared with LaSR media; nevertheless, both media produced statistically significant populations of differentiated cells expressing CD34 (LaSR p = 5.75e−8, TeSR p = 2.88e−6, +Dox vs. −Dox) and VEC (LaSR p = 6.78e−9, TeSR p = 1.18e−6, +Dox vs. −Dox) (Figures 4G, 4H, and S5D). Collectively, these results demonstrated *SOX17* can overcome the presence of pluripotency factors in hPSC media to efficiently produce CD34^+^VEC^+^ cells.

### FGF2 enables an emergent CD31^+^CD34^+^VEC^+^ EP population in a dose-dependent manner

**Figure 5:**
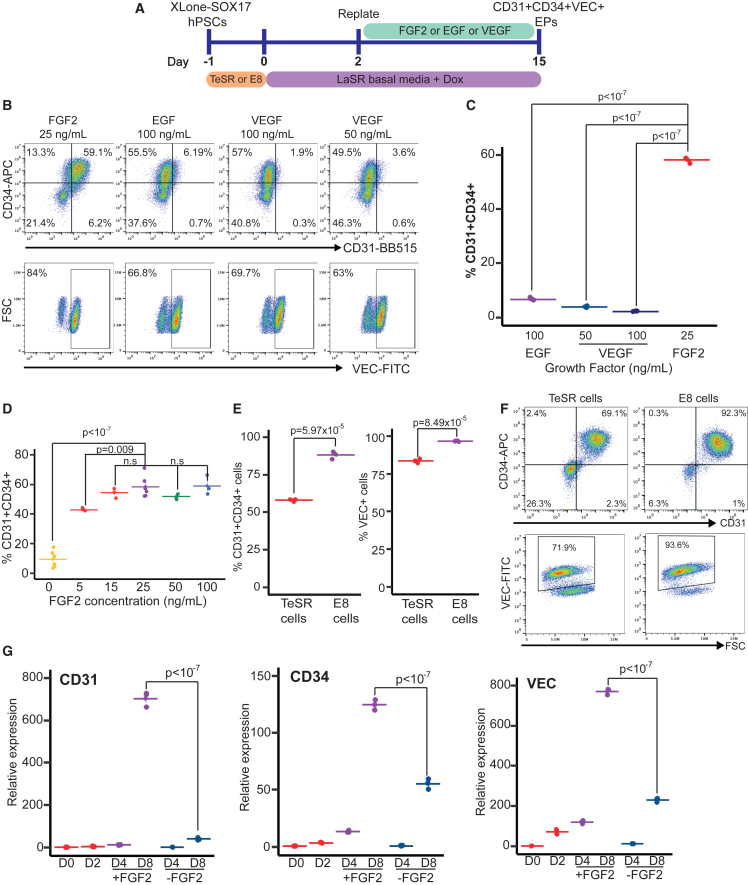
FGF2 supports robust CD31^+^CD34^+^VEC^+^ EP programming via SOX17 (A) Schematic of SOX17-mediated forward programming as supplemented by the indicated growth factors, EGF, VEGF, and FGF2. (B) Flow cytometry results showing the expression of CD31 and CD34 (double stained) and VEC (single stained) for various treatment conditions. (C) Quantification of the CD31^+^CD34^+^ population for different treatment conditions (n = 3, independent experiments). (D) Flow cytometry results for the percentage of CD31^+^CD34^+^ EPs produced by varying FGF2 concentrations, between 0 and 100 ng/mL (n = 6 for 0 and 25 ng/mL, n = 3 for 5, 15, 50, 100 ng/mL, independent experiments). (E and F) Flow cytometry data showing the percentage of CD31^+^CD34^+^ cells obtained from the FGF2-enhanced SOX17 programming protocol, applied to hPSCs cultured in TeSR or E8 media. (n = 3, independent experiments). (G) qPCR analysis for CD31, CD34, and VEC in E8 cells subjected to SOX17-mediated programming, either with or without FGF2, from day 0 to day 8. (n = 3, technical replicates).

While we successfully produced CD34^+^VEC^+^ cells using SOX17 overexpression, these cells showed minimal expression of CD31, another essential EP cell marker. To identify factors that might boost CD31 expression in these cells, we tested the impact of growth factors (epidermal growth factor [EGF], FGF2, and VEGF) known for their roles in endothelial development and support. These factors were introduced to the differentiation culture on day 2 and remained until cell collection and flow cytometry analysis (Figure 5A). By day 5, the addition of FGF2 significantly enhanced CD31 expression, yielding a 64.6% ± 0.7% CD31^+^ cell population (Figures 5B, 5C, and S6A–S6C). Across various concentrations, only FGF2 significantly boosted CD31 expression. In contrast, EGF and VEGF resulted in less than 7% of CD31^+^ cells (Figures 5B and 5C). Furthermore, FGF2 treatment positively influenced other EP markers. In the FGF2-treated group, we observed 71.7% ± 0.5% CD34^+^ and 83.8% ± 0.7% VEC^+^ cells. These percentages are notably higher than those in the EGF- and VEGF-treated samples (Figures 5B and 5C). When we analyzed gene expression using qPCR, we discovered that FGF2 treatment led to a significant increase in the mRNA expression of CD31, CD34, and VEC (Figure S6C). To determine if FGF2’s enhancement of EP programming is dose dependent, we treated cells with 0, 5, 15, 25, 50, and 100 ng/mL FGF2. Remarkably, even a dose of 5 ng/mL had a significant impact, yielding more than 40% CD31^+^CD34^+^ cells (Figure 5D). Concentrations of 25 ng/mL or more were needed to achieve more than 50% CD31^+^CD34^+^ cells. However, increasing the FGF2 concentration beyond this point showed no further benefit (Figure 5D).

We wanted to see if FGF2 could also boost CD31 expression in another iPSC line and, thus, used the XLone-SOX17 6-9-9 cells. After treating these cells with FGF2 along DOX-induced differentiation, there was an increase in the percentage of CD31^+^ cell from 7% to 80% on day 8 (Figures S7A–S7C). This suggests that FGF2 can enhance CD31 expression across different cell lines during SOX17 forward programming.

We next investigated if hPSCs grown in albumin-free E8 media (Chen et al., 2011) could be programmed into EPs using SOX17 and FGF2. To test this, we cultured our XLone-SOX17 H9 cells in E8 media for multiple passages and then treated them with Dox to induce SOX17 expression, along with FGF2 treatment. Impressively, we found that, when hPSCs were cultured in E8 media, a remarkable 92% of them displayed the CD31 and CD34 markers after SOX17 programming. Additionally, more than 93% of these cells showed VEC expression (Figures 5E and 5F). When comparing the programming efficiency of E8 cells with that of cells in TeSR media, the E8 cells showed a significantly higher efficiency (Figure 5E). Furthermore, E8 media is more cost effective than TeSR. Therefore, we have decided to exclusively use E8 media for culturing hPSCs in future EP programming experiments.

Next, we examined the temporal gene expression pattern from day 0 to day 8 during the SOX17-mediated programming for hPSCs cultured in E8, comparing groups with and without FGF2 treatment using qPCR. On day 8, the FGF2-treated groups exhibited a remarkable 705-fold increase in CD31 expression, contrasting with a 40-fold increase in the untreated groups. Similarly, the FGF2-treated samples displayed significantly higher expressions of CD34 (125-fold increase) and VEC (773-fold increase) compared with 55-fold and 232-fold increases, respectively, in the untreated groups (Figure 5G). Together these results demonstrate that FGF2 treatment can significantly increase SOX17-mediated EP programming of hPSCs, yielding more than 90% CD31^+^CD34^+^VEC^+^ cells.

### Differentiation of SOX17-programmed EPs into functional ECs

**Figure 6:**
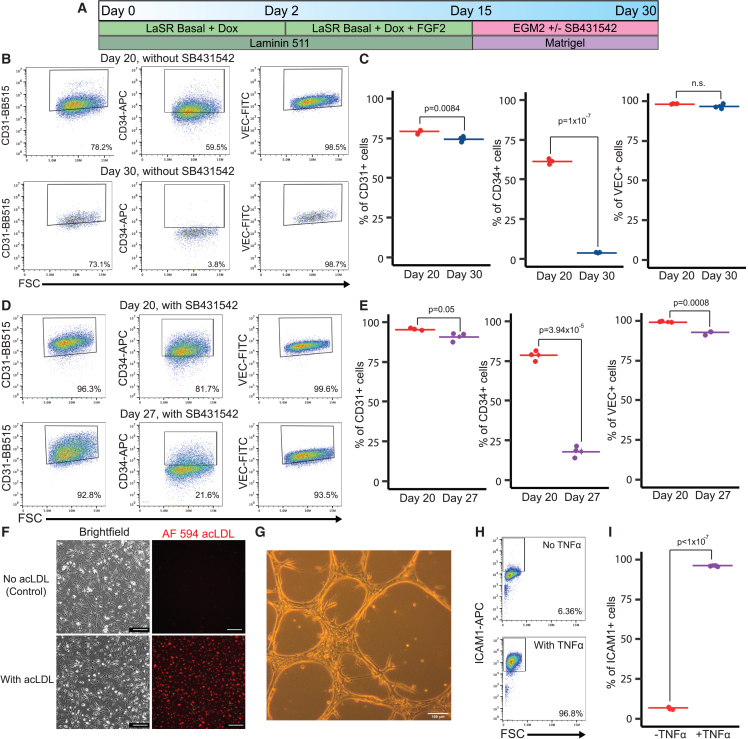
Differentiation of SOX17-programmed EPs into functional ECs (A) Schematic showing differentiation of EPs into ECs with or without SB431542. (B and C) In the absence of SB431542 treatment, flow cytometry was performed for CD31, CD34, and VEC expression in cells cultured in EGM2 on days 20 and 30. These cells were differentiated from day 15 EPs. (C) Quantitative analysis of (B). (n = 3, independent experiments). (D and E) With SB431542 treatment, flow cytometry was performed for CD31, CD34, and VEC expression in cells cultured in EGM2 on days 20 and 27. These cells were differentiated from day 15 EPs. (E) Quantitative analysis of (D). (n = 3, independent experiments). (F) Brightfield and fluorescent images from the acLDL-uptake assay. Cells were either treated with no acLDL (negative control) or 5 μg/mL AF594 acLDL. (G) The resulting ECs were tested for tube-forming ability upon VEGF treatment. Scale bars, 100 μm. (H) Day 15 SOX17-induced ECs were incubated with or without TNF-α for 16 h. ICAM1 expression was measured via flow cytometry. (I) Quantification of data presented in (H). (n = 3, independent experiments).

We next aimed to examine the differentiation of EPs into endothelial cells (ECs) and maintain the expansion of these ECs. Previous studies have demonstrated that the inhibition of transforming growth factor β (TGF-β) with a small molecule SB431542 supports the prolonged expansion of hPSC-derived ECs (James et al., 2010). As a result, we investigated the differentiation of our EPs into ECs and expanded the derived ECs using a commercial EC media (the EGM2 media), with or without the TGF-β inhibitor SB431542 (Figure 6A).

When cultured in EGM2 media without SB431542, cells displayed high levels of CD31 and VEC expression by day 30 (Figures 6B and 6C). Concurrently, these cells lost CD34 expression, suggesting successful differentiation into ECs. However, in the absence of SB431542 treatment, these cells ceased proliferation by day 30. In contrast with the group without SB431542, cells treated with SB431542 displayed persistent proliferation beyond day 30. By day 27, these cells showed a decrease in CD34 expression, akin to the non-treated group. However, they consistently maintained high levels of CD31 and VEC expression. By day 31, 90.9% ± 1.56% of the cells were CD31^+^ and 92.8% ± 0.7% were VEC^+^, while CD34 expression had decreased to 18.1% ± 2.18% (Figures 6D and 6E).

We further assessed the functional capabilities of the ECs by measuring their uptake of Alexa Fluor 594 conjugated acetylated low-density lipoproteins (AF594 acLDL). After adding AF594 acLDL to the media and allowing incubation, fluorescent imaging revealed that ECs on day 30 effectively took up acLDL, indicative of their endothelial function (Figure 6F). Moreover, these ECs formed vascular tubes in the Matrigel matrix when treated with exogenous VEGF (Figure 6G). The endothelium reacts to inflammatory mediators, such as tumor necrosis factor α (TNF-α), by increasing the expression of adhesion molecules like intercellular cell adhesion molecule 1 (ICAM1), which is associated with the capture of circulating leukocytes. In the absence of TNF-α treatment, SOX17-induced ECs exhibit low levels of ICAM-1 expression, but TNF-α treatment significantly enhances ICAM1 expression in the SOX17-induced ECs (Figures 6H and 6I).

**Figure 7:**
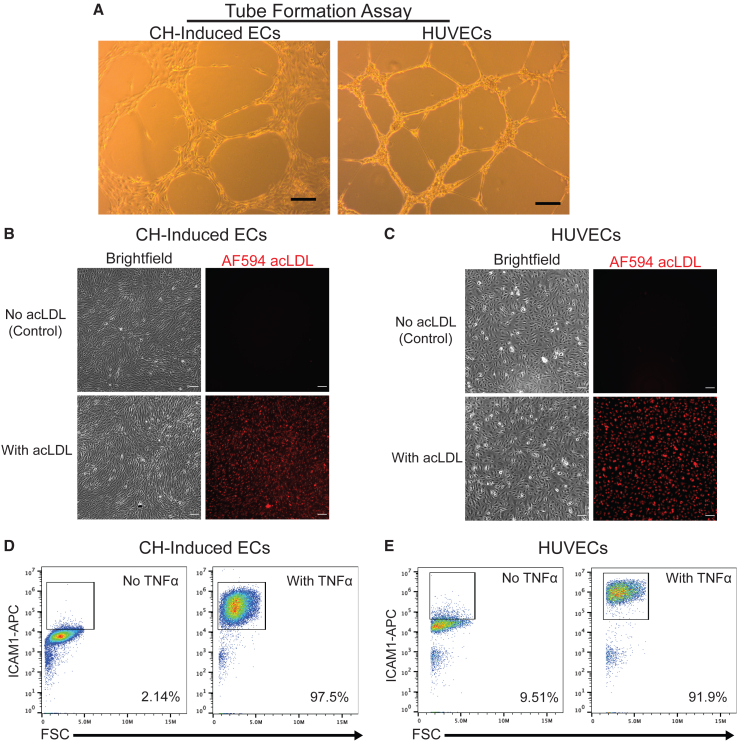
Functional characterization of CH-induced ECs and HUVECs (A) CH-induced ECs (left) and HUVECs (right) were characterized for tube-forming ability upon VEGF treatment. Scale bars, 100 μm. (B and C) Brightfield and fluorescent images from the acLDL-uptake assay. CH-induced ECs (B) and HUVECs (C) were treated with or without Alexa Fluor 594 acLDL (AF594 acLDL). Scale bars, 100 μm. (D and E) CH-induced ECs (D) and HUVECs (E) were incubated with or without TNF-α for 16 h. ICAM1 expression was quantified via flow cytometry.

To further evaluate the functional characteristics of SOX17-induced ECs, we introduced two control groups into our study: ECs differentiated from hPSCs through CHIR99021 treatment (referred to as CH-induced ECs)(Lian et al., 2014) and human umbilical vein ECs (HUVECs). Employing three distinct functional assays—tube formation on Matrigel, acLDL uptake, and responsiveness to the inflammatory factor TNF-α—we assessed the performance of these control cell types. Our data reveal that both control cell types are capable of tube formation, acLDL uptake, and exhibit upregulation of ICAM1 in response to TNF-α treatment (Figure 7). The data collectively demonstrate that SOX17-induced ECs exhibit comparable functional properties to both CH-induced ECs and HUVECs.

In summary, our findings suggest that cells programmed with SOX17 and FGF2 can differentiate and proliferate in EC media when using a TGF-β inhibitor. These derived ECs not only exhibit acLDL uptake and upregulation of ICAM1 expression upon TNF-α treatment, but also have the ability to form vascular tubes on the Matrigel matrix.

## Discussion

Forward programming has proven to be an effective strategy for the generation of various somatic cell types (Sugimura et al., 2017). SOX17 plays a pivotal role in governing the specification of the endothelial lineage and endothelial regeneration (Goveia et al., 2014; Liu et al., 2019; Trinh et al., 2023). Furthermore, SOX17 expression has been observed in many other cells, such as definitive endoderm cells (Jiang et al., 2021) and primordial germ cells (Irie et al., 2015). In this study, we provide the first evidence demonstrating that overexpressing *SOX17* in hPSCs cultured in LaSR basal medium (Lian et al., 2014) is sufficient to produce CD34^+^VEC^+^ cells, eliminating the need of other small molecules and growth factors. Furthermore, when FGF2 is introduced during the SOX17-mediated programming, it results in the generation of more than 90% CD34^+^VEC^+^CD31^+^ EPs. These progenitors can then further differentiate into functional ECs under the right conditions.

Our scRNA-seq data, derived from EPs generated using our small molecule Wnt activation EP protocol, underscored the differential expression of SOXF factors. However, only SOX17 enhanced the differentiation efficiency when overexpressed during the EP differentiation. Despite belonging to the same SOXF sub-family, SOX7, SOX17, and SOX18 exhibit distinct interactions with various partner proteins, leading to the induction of unique gene expression patterns. For example, Sox7 demonstrates the capability to induce the expression of Fgf3 (Murakami et al., 2004) and VEC (Costa et al., 2012; Doyle et al., 2019). Conversely, SOX18 exhibits the ability to induce the expression of MAP4K4 (Moustaqil et al., 2018), CLDN5 (Fontijn et al., 2008), and Prox1 (Klaus et al., 2016), while SOX17 elicits the expression of CSNK1A1 (Kuo et al., 2014), MBP (Sohn et al., 2006), and Hnf1B (Hudson et al., 1997). Interestingly, all three SOXF factors enhanced VEC expression, aligning with studies revealing SOX17 and SOX7’s ability to activate VEC expression (Costa et al., 2012; Nakajima-Takagi et al., 2013). Given the SOXF subgroup’s conserved nature and analogous binding sites, it is plausible that all three exert a similar influence on VEC transcription in humans. Notably, the expression of CD31 in the context of SOX17 forward programming only became apparent after the introduction of FGF2 from day 2 onward.

To further investigate SOX17’s role, we employed Cas13d in loss-of-function studies. Results indicated that SOX17 knockdown significantly restricts hPSCs differentiation into EPs, mirroring previous studies (Clarke et al., 2013; Jung et al., 2021; Kim et al., 2016; Nakajima-Takagi et al., 2013). Having confirmed the functional importance of SOX17 by loss of function, we went on to show that forward programming by *SOX17* overexpression is sufficient to obtain CD34^+^VEC^+^ cells from multiple hPSC lines in a variety of different culture media, including hPSC media. We optimized forward programming conditions to find the ideal temporal window and level for transgene expression. After evaluating growth factors to enhance CD31 expression, we found that, by combining FGF2 treatment with SOX17 forward programming, we can consistently achieve a high purity of CD31^+^CD34^+^VEC^+^ EPs, with yields exceeding 90%. These EPs can further differentiate into functional ECs that uptake acLDL and form vascular tubes in the Matrigel matrix.

In summary, this is the first documented instance of using SOX17 for hPSC forward programming into EPs. It also uniquely showcases a single TF facilitating endothelial commitment without relying on typical mesodermal induction or standard growth factors like activin A, BMP4, Wnt ligands or VEGF. These insights elevate SOX17’s significance in human endothelial development and warrant further exploration. Although extensive research has explored endothelial fate and development, the genetic intricacies of human endothelial development remain elusive. We anticipate that our current and forthcoming discoveries will enrich our comprehension of human endothelial development and pave the way for improved manufacturing of therapeutically relevant cells.

## Experimental procedures

### Resource availability

#### Lead contact

Further information and requests for resources and reagents should be directed to and will be fulfilled by the corresponding author Dr. Xiaojun Lance Lian (Lian@psu.edu).

#### Materials availability

All plasmids generated from this paper will be available at www.addgene.org/Xiaojun_Lian/.

#### Data and code availability

• High-throughput sequencing data obtained in this study have been submitted to GEO and are available under the accession number GEO: GSE161408.
• This paper does not report original code.
• Any additional information required to re-analyze the data reported in this paper is available from the corresponding author (Lian@psu.edu) upon request.

#### Maintenance of hPSCs

Human embryonic stem cells (H9, H9 SOX17-mCherry, and H1 OCT4-GFP) and iPSCs (6-9-9) were maintained on either Matrigel (Corning) or iMatrix-511 silk (Iwai North America) coated plates in TeSR (Stemcell Technologies), LaSR, or E8 media according to previously published methods (Bao et al., 2015; Lian et al., 2013). All the transgenic XLone hPSCs were maintained with 10 μg/mL blasticidin (Sigma) to prevent construct silencing. All drugs were removed upon initiating differentiation or forward programming. Cells were routinely tested to ensure mycoplasma free culture conditions using an established PCR-based detection method. Cell line details are included in Table S2.

#### EP differentiation of hPSCs via CH and VEGF

EP differentiation of hPSCs was initiated when hPSCs seeded on Matrigel or iMatrix-511 silk coated plates reached 60% confluence. Differentiation was performed according to our previously published methods (Bao et al., 2015; Lian et al., 2014). Briefly, at day 0, cells were treated with 6 μM CHIR99021 (Cayman Chemical) for 48 h in LaSR Basal media, which consists of Advanced DMEM/F12, 2.5 mM GlutaMAX and 60 μg/mL ascorbic acid, with media refreshed after 24 h. From day 2 to day 5, cells were maintained in LaSR basal medium with 50 ng/mL VEGF. Analysis was performed on day 5.

#### SOX17-mediated endothelial programming of hPSCs

For forward programming experiments, XLone-SOX17 hPSCs were cultured on Matrigel or iMatrix-511 coated plates. When cells reached 10% confluence, Dox was added from day 0 to 3 at 1 μg/mL and from day 3 to 5 at 5 μg/mL. Cells were re-plated on iMatrix-511 coated plates on day 2 by dissociation with Accutase (Innovative Cell Technologies) for 5 min at 37°C, pelleting, and resuspension in the day 2 media with 5 μM Y27632. The seeding density on day 2 should be 18,000 cells/cm^2^. From day 2, 25 ng/mL FGF2 should be added into the media to enhance EP programming. Media should be changed every day, and the cells grew without further passaging after day 2 until harvesting for analysis.

#### Differentiation of EPs into ECs

Day 15 EPs were seeded onto Matrigel coated plates at a seeding density of 5,000/cm^2^. EGM2 media (Lonza) supplemented with 10 μM SB413542 (Cayman Chemicals) were used to culture the cells. A ROCK inhibitor GSK269962A (Selleck Chemicals) was used for the first 24 h following passaging. When cell culture reach more than 90% confluency, cells will be passaged via Accutase and replated at the same cell density in EGM2, supplemented with SB431542 and GSK269962A.

#### scRNA-seq

H9 cells were differentiated using Wnt activation EP differentiation protocol. On day 5 of differentiation, cells were treated with Accutase for 10 min. Single cells were counted and resuspended in PBS with 0.04% BSA. A cell strainer was used to get rid of debris and clumps of cells. The single cell library was constructed using the Chromium Next GEM Single Cell 3′ protocol. Then the library was sequenced on a NextSeq 550 equipment with the High Output 150 cycle kit. scRNA-seq data were processed through the 10× Genomics Cell Ranger pipeline to generate count matrices. These count matrices were then analyzed using Seurat version 3.2.1 (Butler et al., 2018; Stuart et al., 2019). Briefly, quality control filters were applied to sort out dying or dead cells and multiplets. The gene expression for each cell was then normalized by the total expression, scaled, and log transformed. A linear transform was then applied to the data prior to dimensional reduction via principal-component analysis. Statistical (JackStraw) and heuristic (elbow plot) strategies were used to determine the number of principle components to include.

#### Immunostaining

Cells were fixed with 4% formaldehyde (Sigma) for 15 min at room temperature. Then, cells were washed three times with DPBS and then blocked for 1 h at room temperature with DPBS with 0.4% Triton X-100 and 5% non-fat dry milk (BioRad). Cells were then stained with primary antibodies (Table S3) in DPBS with 0.4% Triton X-100 and 5% non-fat dry milk for 2 h. Then cells were washed with DPBS three times and incubated with secondary antibodies (Table S3) for 1 h. Cells were washed three times with DPBS. Nuclei were stained with Hoechst 33342 (Thermo Fisher Scientific). A Nikon Ti Eclipse epifluorescence microscope was used for image capture and analysis. Fiji and MATLAB were used for further analyses and quantification.

#### Flow cytometry analysis

For staining and analysis of fixed cells, after dissociation with TrypLE Express (differentiated cells) or Accutase (hPSCs), cells were pelleted and resuspended in DPBS with 1% formaldehyde for 30 min at room temperature. Cells were pelleted and washed three times with DPBS. Cells were stained with primary antibodies in DPBS with 0.1% Triton X-100 and 0.5% BSA for 2 h at room temperature. Then cells were stained with secondary antibodies for 30 min at room temperature in the dark. Then cells were pelleted and washed three times with DPBS with 0.5% BSA before analysis. For staining and analysis of live cells, cells were dissociated with TrypLE Express (differentiated cells) or Accutase (hPSCs) and pelleted. Cells were then resuspended in DPBS with 0.5% BSA and the appropriate amount of conjugated primary antibodies. Then cells were incubated at room temperature for 30 min in the dark. Cells were pelleted and washed with DPBS with 0.5% BSA. Data were collected on a BD Accuri C6 Plus flow cytometer and analyzed using FlowJo. Gating was based on the corresponding untreated or secondary antibody-stained cell control.

#### Western blotting

Cells were washed with DPBS and lysed with Mammalian Protein Extraction Reagent (Thermo Fisher Scientific) with 1× Halt’s Protease and Phosphatase (Thermo Fisher Scientific) by incubation for 3 min. Cell lysate was collected and stored at −80°C until used. Samples were mixed with Laemmli sample buffer (BioRad) at a working concentration of 1× and incubated at 97°C for 5 min. Samples were loaded into a pre-cast MP TGX stain-free gel (BioRad) and run at 200 V for 30 min in 1× Tris/Glycine/SDS buffer (BioRad). Protein was transferred to a polyvinylidene fluoride membrane using a *Trans*-blot Turbo Transfer System (BioRad). The membrane was blocked for 30 min at room temperature in 1× TBST with 5% dry milk. The membrane was incubated overnight at 4C with primary antibodies, followed by 1 h at room temperature with secondary antibodies in 1× TBST with 5% dry milk. The membrane was washed between each antibody exposure with 1× TBST. Chemiluminescence was activated using Clarity Western ECL Substrate (BioRad) and the blot was imaged using a ChemiDoc Touch Imaging System and Image Lab software (BioRad). Blots were analyzed using Fiji software.

#### qPCR

RNA was extracted from cells using a Direct-zol RNA MiniPrep Plus Kit (Zymo Research). A Maxima First Strand cDNA Synthesis kit (Thermo Fisher Scientific) was used to generate cDNA. A BioRad CFX Connect system was used for performing qPCR with the PowerSYBR Green PCR Master Mix (Thermo Fisher Scientific) and primers (Table S4). Data were analyzed by the ΔΔ cycle threshold (ΔΔC_t_) method, where target C_t_ values were normalized to glyceraldehyde-3-phosphate dehydrogenase (GAPDH) C_t_ values and fold changes in target gene expression were determined by comparing with day 0 samples. Each sample was run in triplicate. In the event that no measurable expression was detected, the relative expression to GAPDH was set to zero.

#### Generation of XLone-SOXF hPSC lines

The open reading frame for human SOX17 was PCR amplified using GoTaq Master Mix (Promega) from the PB-TRE3G-SOX17 plasmid. The amplicon was gel purified and ligated into XLone, which was linearized using restriction enzymes KpnI and SpeI (New England Biolabs), using In-Fusion ligase (TaKaRa Bio). XLone-SOX7 and XLone-SOX18 were cloned into XLone by Genewiz. To generate transgenic cell lines, hPSCs were dissociated with Accutase for 10 min at 37°C and pelleted. The cell pellet was resuspended in 100 μL PBS with 8 μg plasmid DNA, including 3 μg EF1a-hyPBase and 5 μg XLone-SOX17 (Table S5). The mixture was transferred to a cuvette and nucleofected using the CB150 program on the Lonza 4D Nucleofector. All plasmid DNA used was prepared using an Invitrogen PureLink HiPure Plasmid Filter Midiprep Kit. Cells were plated at a high density with 5 μM Y27632. Successfully modified cells were purified using media supplemented with 30 μg/mL blasticidin. Upon achieving a relatively pure population, cells were maintained in media containing 10 μg/mL blasticidin. All plasmids generated have been submitted to Addgene.

#### acLDL uptake assay

After a minimum of two passages in the EC media, cells underwent an additional passage into EGM2 supplemented with SB431542. Subsequently, the media was replaced with fresh EGM2 containing 5 μg/mL AF594-labeled acLDL (Invitrogen) and the plate was incubated at 37°C in the dark for 2 h. After incubation, the wells were washed three times with PBS and replenished with fresh EGM2. Cells were then allowed to recover at 37°C for 5 min before imaging with fluorescence microscopy.

#### Vascular tube formation assay

To evaluate capillary structure formation, 1 × 10^5^ ECs in 0.4 mL EGM2 medium supplemented with 50 ng/mL VEGF were plated into one well of a 24-well tissue culture plate precoated with 250 μL Matrigel. After 24 h of incubation, tube formation was inspected using light microscopy.

#### Statistics

Experiments were performed in triplicate. Data obtained from multiple experiments or replicates are shown as the mean ± SEM. Where appropriate, one or two tailed Student’s t test or ANOVA was utilized (alpha = 0.05) with a Bonferroni or Tukey’s post hoc test where appropriate. Data were considered significant when p < 0.05. Statistical tests were performed using MATLAB or GraphPad Prism.

## Supplemental information

Document S1. Figures S1–S7 and Tables S2–S5

Table S1. Top 100 differentially expressed genes in the 5 clusters of cells on day 5

Document S2. Article plus supplemental information
