# Supplementary material for: Direct programming of human pluripotent stem cells into endothelial progenitors with SOX17 and FGF2
Source: Stem Cell Reports. 2024 Mar 21;19(4):579–95. doi: 10.1016/j.stemcr.2024.02.006 (PMC11096437; doi:10.1016/j.stemcr.2024.02.006)
Supplement: Document S1. Figures S1–S7 and Tables S2–S5 [file mmc1.pdf]

Stem Cell Reports, Volume *19*

Supplemental Information

Direct programming of human pluripotent stem cells into endothelial progenitors with SOX17 and FGF2

Michael W. Ream, Lauren N. Randolph, Yuqian Jiang, Yun Chang, Xiaoping Bao, and Xiaojun Lance Lian

Supplemental figures and legends

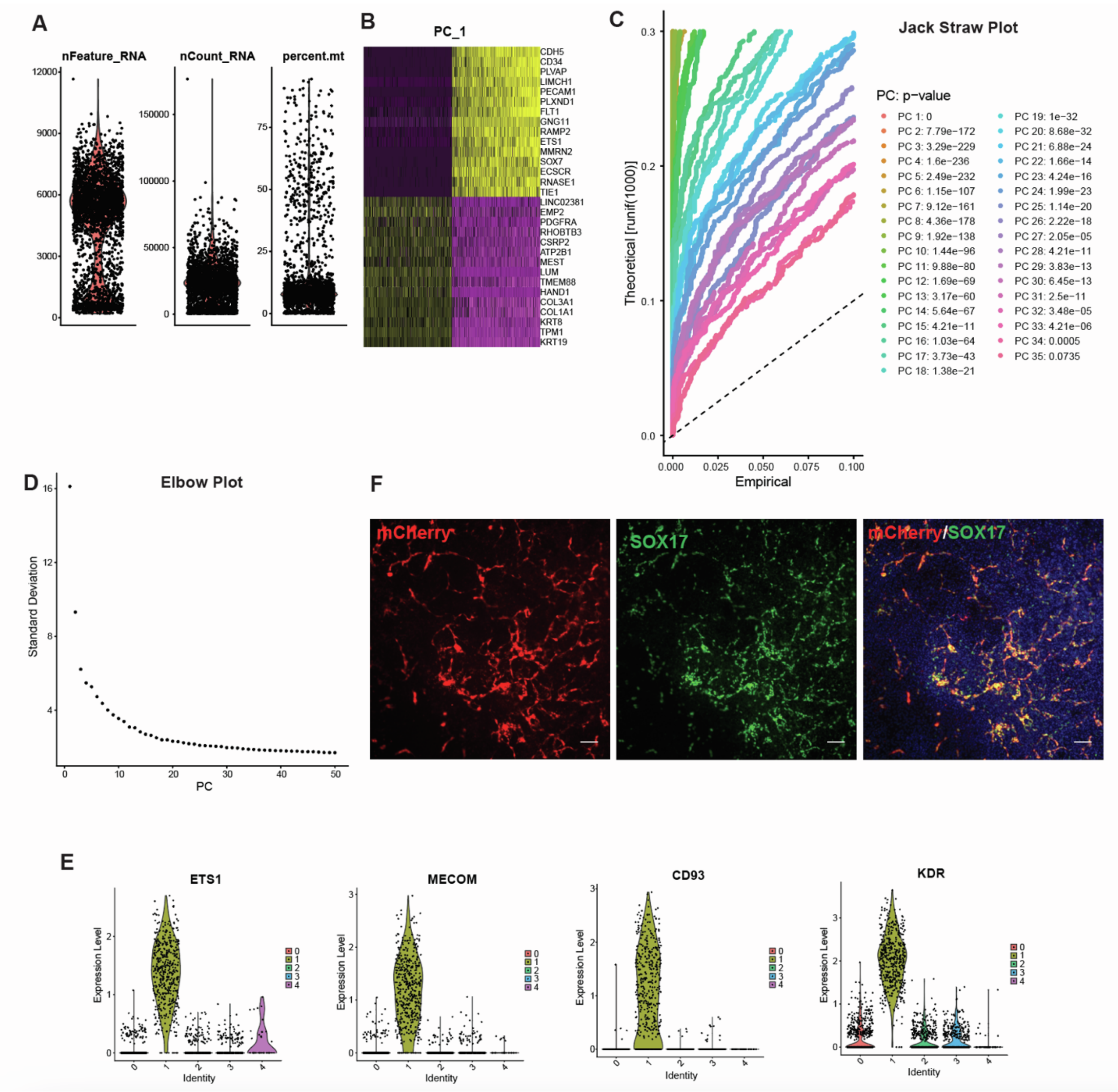

Fig. S1. Single cell RNA sequencing data analysis and validation. A) Violin plots indicating the number of genes, counts, and percentage of the reads mapped to mitochondrial genes prior to applying filters. **B)** Heat map for principal component 1 from PCA. **C)** Jack Straw plot for the first 50 principal components. **D)** Elbow plot for the first 50 principal components. **E)** Violin plots identifying ETS1, MECOM, CD93, and KDR expression in cluster 1. **F)** Immunofluorescence showing mCherry reporter accuracy by comparison with antibody staining of SOX17. Scale bars are 100 μm.

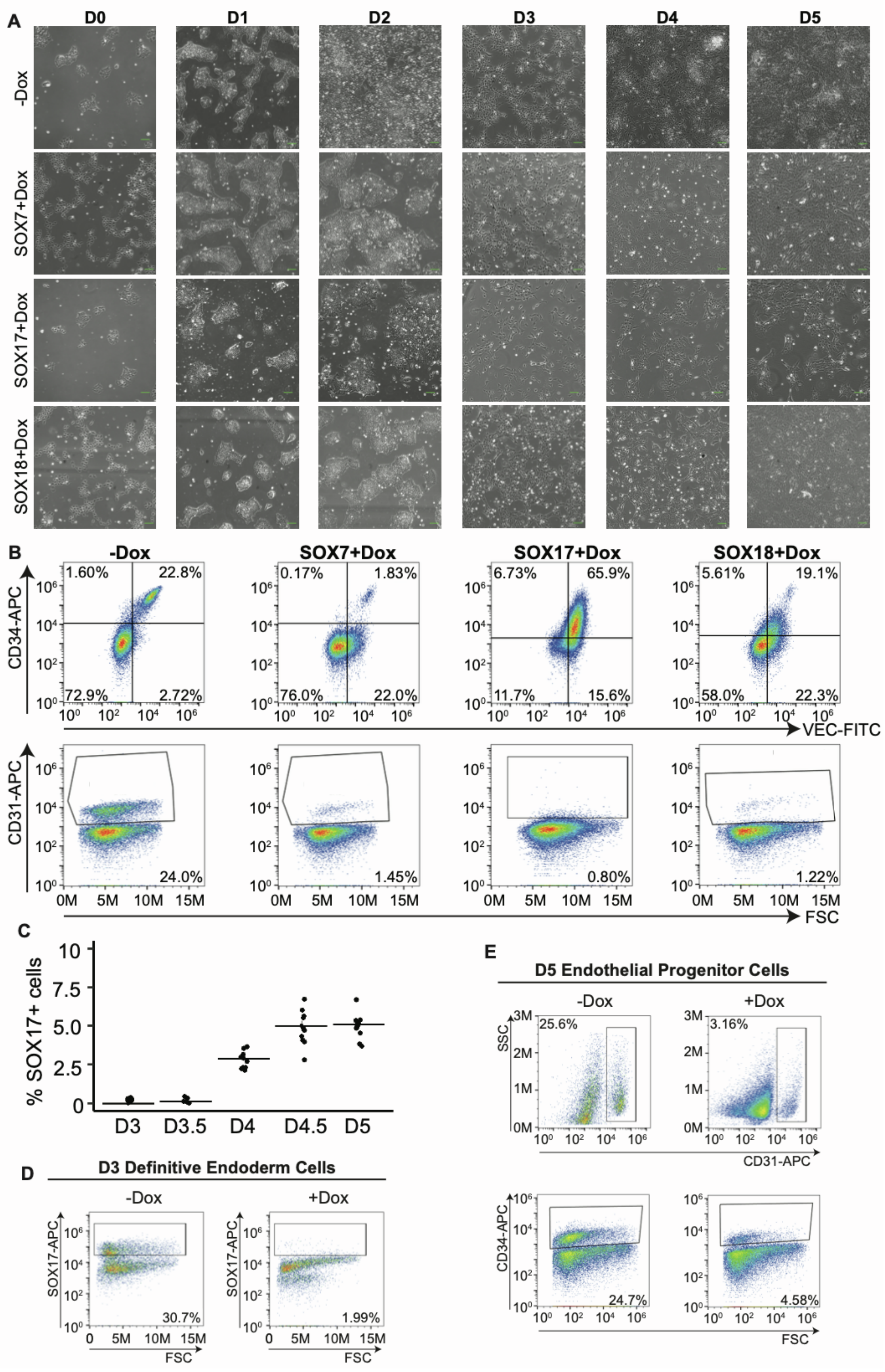

A
D0
D1
D2
D3
D4
D5
-Dox
SOX7+Dox
SOX17+Dox
SOX18+Dox
B
-Dox
SOX7+Dox
SOX17+Dox
SOX18+Dox
CD34-APC
VEC-FITC
1.60%
22.8%
72.9%
2.72%
0.17%
1.83%
76.0%
22.0%
6.73%
65.9%
11.7%
15.6%
5.61%
19.1%
58.0%
22.3%
CD31-APC
FSC
24.0%
1.45%
0.80%
1.22%
C
% SOX17+ cells
D3
D3.5
D4
D4.5
D5
D
D3 Definitive Endoderm Cells
-Dox
+Dox
SOX17-APC
FSC
30.7%
1.99%
E
D5 Endothelial Progenitor Cells
-Dox
+Dox
SSC
CD31-APC
25.6%
3.16%
CD34-APC
FSC
24.7%
4.58%

Fig. S2. CH-induced differentiation with SOXF overexpression and Cas13d-mediated SOX17 knockdown. A) Brightfield images of control, XLone-SOX7, XLone-SOX17, and XLone-SOX18 H9 cells differentiated to day 5 EP cells with and without Dox. Scale bars are 100 μm. **B)** Representative flow cytometry plots analyzing CD34, VEC, and CD31 expression in day 5 cells (n=3). **C)** Quantification of the percentage of SOX17^{+} cells during CH-induced EP differentiation. (n=10, independent experiments). Error bars represent standard error of the mean. **D)** Flow analysis of day 3 definitive endodermal cells differentiated with and without Dox treatment to validate efficacy of Cas13d knockdown of SOX17. **E)** Representative flow analysis plots showing CD31 and CD34 expression in day 5 endothelial progenitors differentiated with and without Dox treatment (n=3).

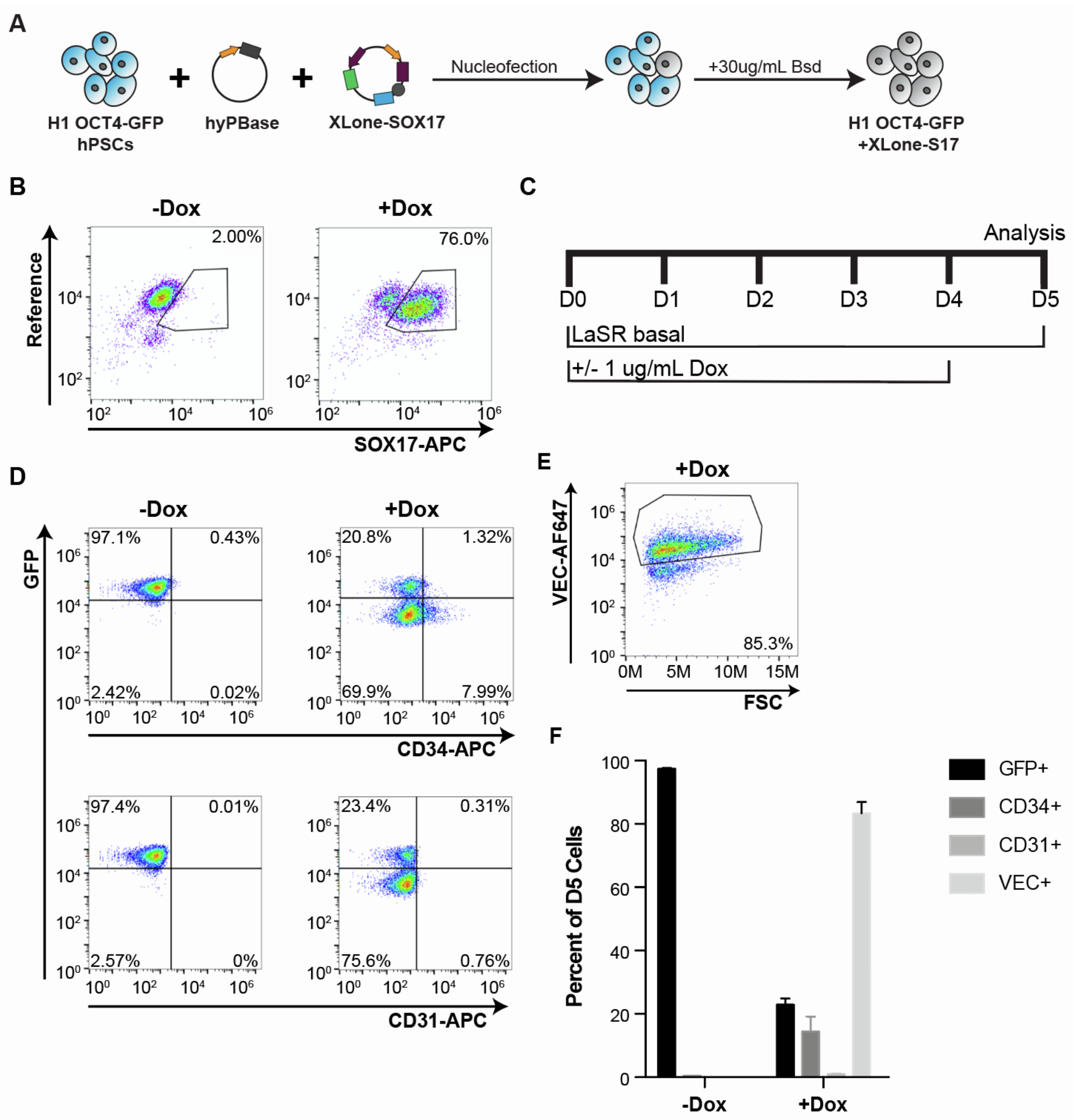

Fig. S3. Forward programming of XLone-SOX17 H1 OCT4-GFP cells. A) Schematic showing the generation of XLone-SOX17 H1 OCT4-GFP cells. **B)** Flow analysis showing SOX17 expression with and without 24 hours of Dox treatment. **C)** Experimental schematic of forward programming protocol. **D and E)** Representative flow analysis of CD34, CD31, GFP (D), and VEC (E) expression for day 5 forward programmed cells (n=3). **F)** Quantification of flow cytometry data (n=3, independent experiments). Error bars represent standard error of the mean.

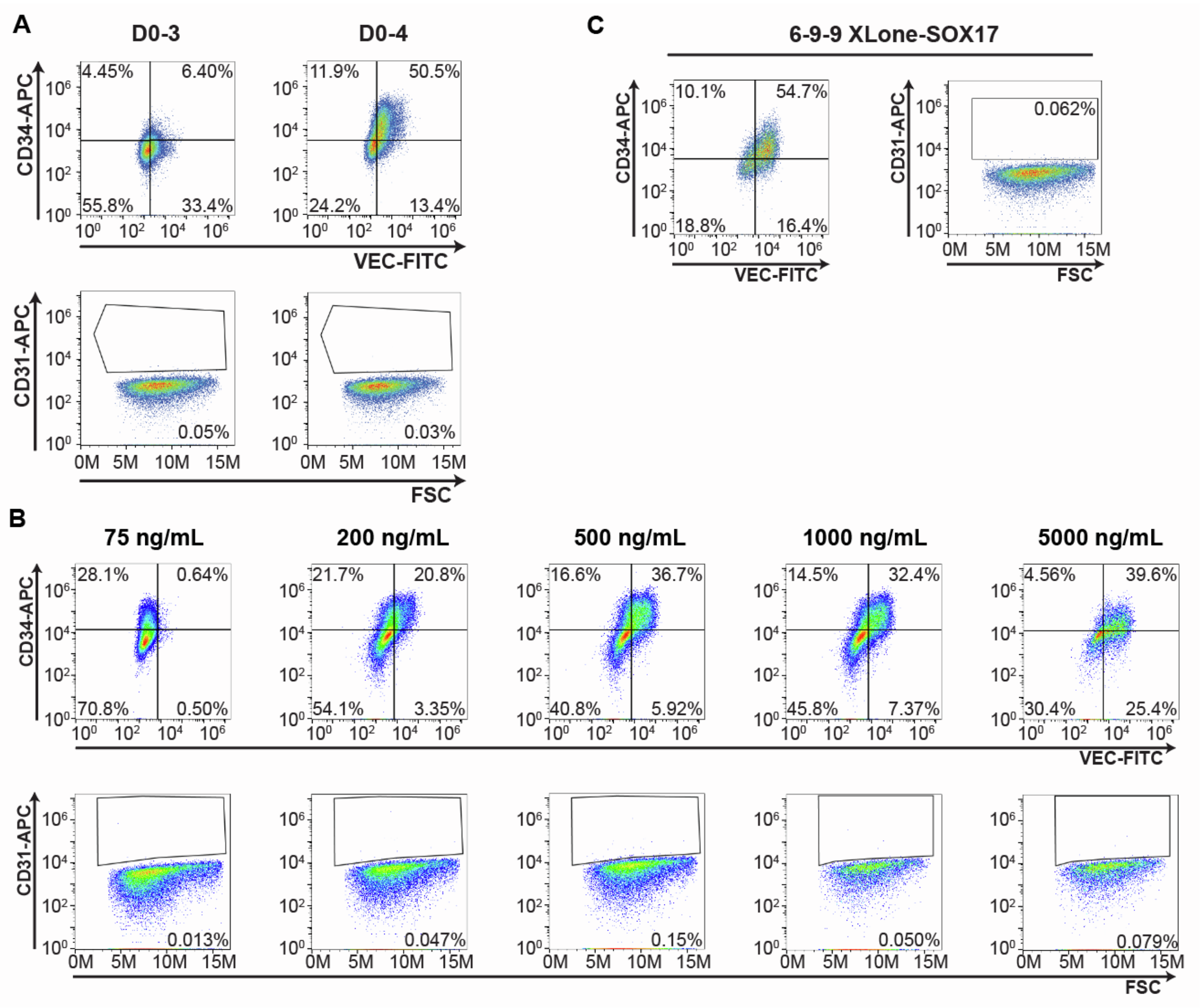

Fig. S4. SOX17 forward programming temporal and concentration optimization. A) Representative flow cytometry analysis for CD34, CD31, and VEC of day 5 forward programmed cells treated with Dox for 3 or 4 days (n=3). **B)** Representative flow cytometry analysis of CD34, CD31, and VEC for SOX17 day 5 forward programmed cells treated with varied concentrations of Dox for 5 days (n=3). **C)** Flow cytometry analysis for CD34, CD31, and VEC of XLone-SOX17 6-9-9 cells day 5 forward programmed cells treated with Dox for 5 days and passaged on day 2.

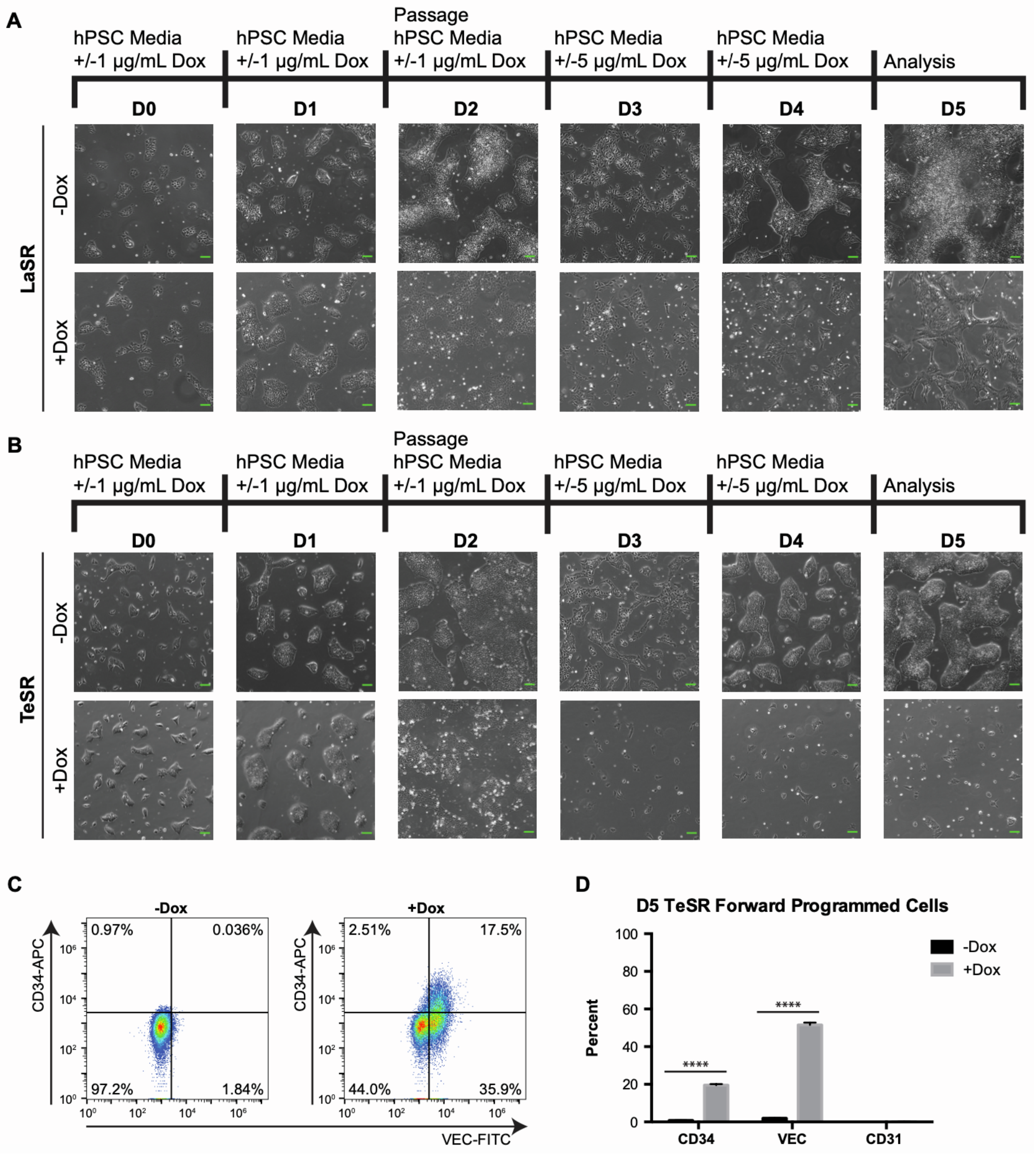

Fig. S5. SOX17 forward programming in hPSC media. A) Bright field images of cells forward programmed with or without SOX17 overexpression in LaSR hPSC media. Scale bars are 100 μm. **B)** Bright field images of cells forward programmed with or without SOX17 overexpression in TeSR hPSC media. Scale bars are 100 μm. **C)** Representative flow cytometry analysis of CD34 and VEC expression in day 5 cells forward programmed in TeSR hPSC media (n=3). **D)** Quantification of flow cytometry analysis for CD34, CD31, and VEC in day 5 cells forward programmed in TeSR hPSC media(n=3, independent experiments). **** indicates $p < 0.0001$. Error bars represent standard error of the mean.

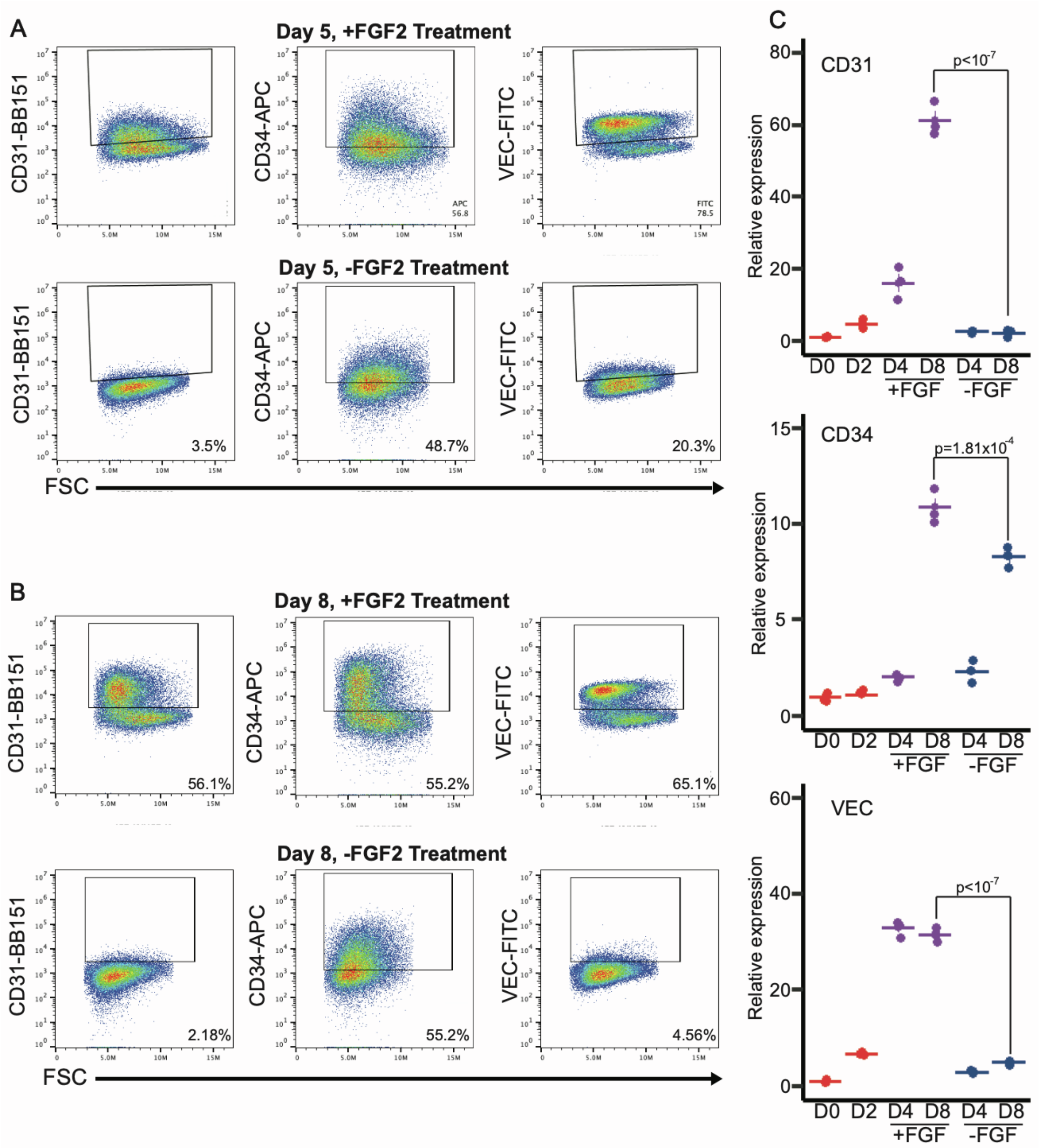

Fig. S6. FGF2 enhanced SOX17-mediated EP programming. A-B) Flow cytometry data showing the expression of CD31, CD34, and VEC on day 5 (A) or day 8 (B) of SOX17 programmed cells treated with FGF2 (top) or not treated with FGF2 (bottom). **C)** qPCR data for CD31, CD34, and VEC expression over the 8 days, with timepoints taken every two days, during SOX17-mediated EP programming with and without FGF2 treatment. (n=3, technical replicates)

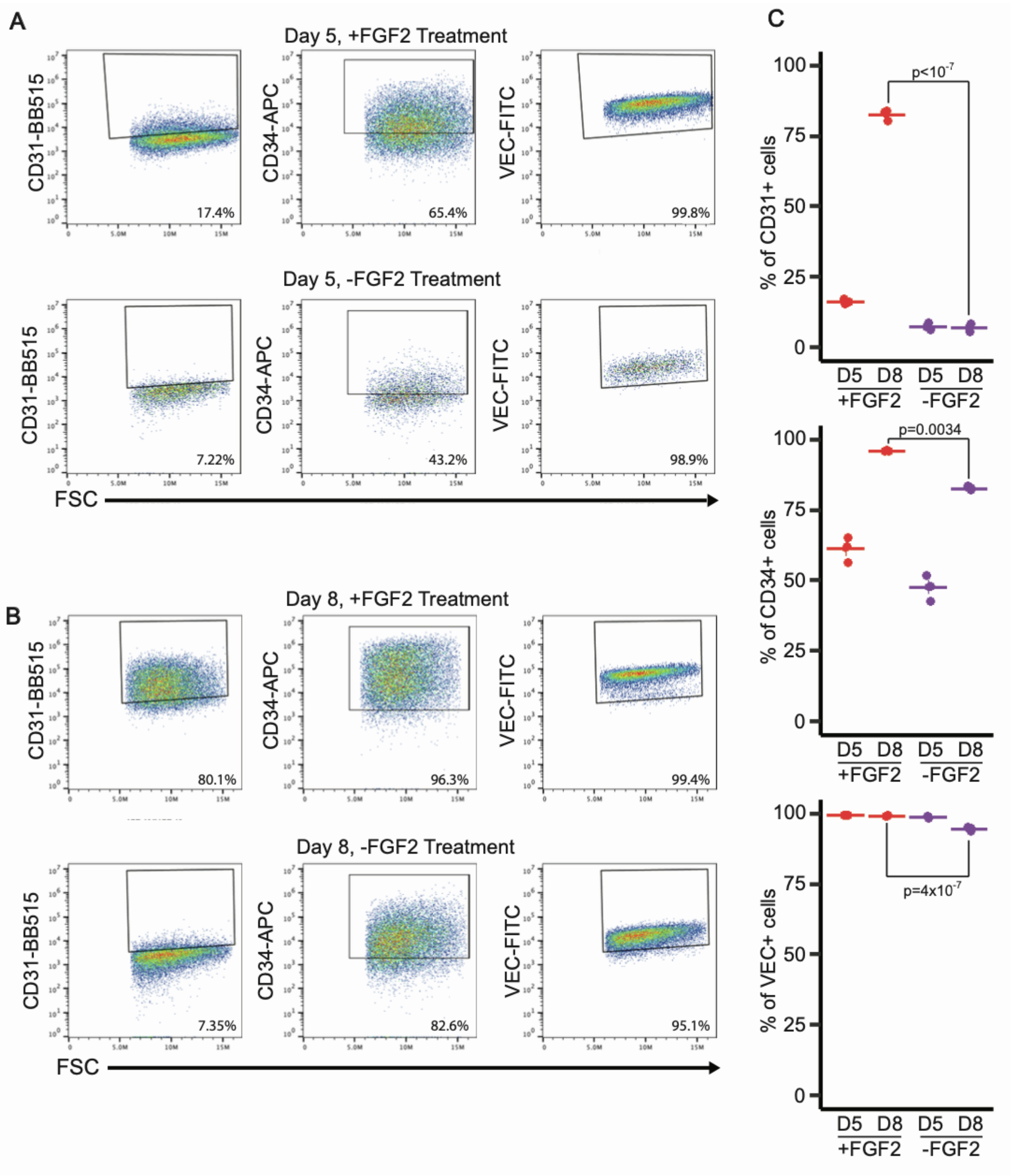

Fig. S7. FGF2 enhanced SOX17-mediated EP programming in XLone-SOX17 6-9-9 iPSC cells. A-B) Flow cytometry data showing the expression of CD31, CD34, and VEC on day 5 (A) or day 8 (B) of SOX17 programmed cells treated with FGF2 (top) or not treated with FGF2 (bottom) in XLone-SOX17 6-9-9 cells. **C)** Quantification of panel (A) and (B). (n=3, independent experiments)

Table S1. Top 100 differentially expressed genes in the 5 clusters of cells on day 5.

Table S2. Cell Lines used in this study

Cell Line Name	Source	Sex	Cell Type
H9	WiCell	F	hESC
6-9-9	WiCell	M	Human iPSC
H9 SOX17-mCherry	These cells were a generous gift from Ed Stanley.	F	Genetically modified knock-in reporter hESC
H1 OCT4-GFP	WiCell	M	Genetically modified knock-in reporter hESC

Table S3. Antibodies used in this study

Antibody	Source/Host and Isotype/Clone/Catalog#	Application
SOX17-APC	R&D Systems/ goat IgG/ IC1924A	1:50 (IF/FC)
VE-Cadherin	Santa Cruz/ mouse IgG_1/ Clone: F-8/ sc-9989	1:100 (IF/FC) 1:1000 (WB)
CD144-FITC	MACS/ recombinant human IgG_1/ Clone REA199/ 130-100-742	1:100 (FC)
CD34-FITC	MACS/ mouse IgG2a / Clone AC136/ 130-113-178	1:100 (FC)
CD34-APC	MACS/ mouse IgG2a / Clone AC136 / 130-113-176	1:100 (FC)
SOX17	R&D Systems/ rabbit IgG/ IC1924A	1:20000 (WB)
β-actin-HRP	Cell Signaling Technology/ rabbit/ Clone: 13E5/ 5125S	1:20000 (WB)
CD31-APC	MACS/ mouse / Clone:AC128 /130-092-652	1:100 (IF/FC)
CD73-PE	Biolegend/ mouse IgG1 / Clone:AD2 /344003	1:100 (FC)
CD45-APC	BIolegend/ Clone 2D1 / 368511	1:20 (FC)
CD44-FITC	Biolegend/ Clone BJ18 / 338803	1:20 (FC)
Secondary Antibody	Alexa 488 Goat anti Ms IgG/ A-11029	1:1000
Secondary Antibody	Anti-mouse IgG HRP-linked/ CST 7076S	1:1000
Secondary Antibody	Goat IgG HRP-conjugated/ R&D Systems HAF017	1:1000
Secondary Antibody	Alexa 647 Goat anti Rb IgG/ A-21244	1:1000
Secondary Antibody	Alexa 647 Goat anti Ms IgG/ A-21235	1:1000

Table S4. qPCR primers in this study

Primer	Sequence
GAPDH_FWD	5'-GTGGACCTGACCTGCCGTCT-3'
GAPDH_REV	5'-GGAGGAGTGGGTGTCGCTGT-3'
OCT4_FWD	5'-CAGTGCCCGAAACCCACAC-3'
OCT4_REV	5'-GGAGACCCAGCAGCCTCAAA-3'
SOX2_FWD	5'-CAAGATGCACAACTCGGAGA-3'
SOX2_REV	5'-GTTCATGTGCGCGTAACTGT-3'
SOX17_FWD	5'-GGCGCAGCAGAATCCAGA-3'
SOX17_REV	5'-CCACGACTTGCCCAGCAT-3'
CD31_FWD	5'-GCTGACCCTTCTGCTCTGTT-3'
CD31_REV	5'-TGAGAGGTGGTGCTGACATC-3'
VEC_FWD	5'-GTGTTCACGCATCGGTTGTT-3'
VEC_REV	5'-CAAATGTGTACTTGGTCTGGGTG-3'
CD34_FWD	5'-CCTAAGTGACATCAAGGCAGAA-3'
CD34_REV	5'-GCAAGGAGCAGGGAGCATA-3'

Table S5. Plasmids used in this study

Plasmid	Source	Addgene #
XLone	This plasmid was generated in the Lian Lab.	96930
XLone-SOX17	This plasmid was generated in the Lian Lab.	135764
XLone-SOX7	This plasmid was generated in the Lian Lab.	163823
XLone-SOX18	This plasmid was generated in the Lian Lab.	163822
XLone-Cas13d-SOX17-gRNA	This plasmid was generated in the Lian Lab.	155184
PB-TRE3G-SOX17	This plasmid was a gift from David Vereide.	104541
EF1a-hyPBase	This plasmid was a gift from Pentao Liu.	NA
